# *EGFR*基因20外显子插入突变在非小细胞肺癌的研究及其进展

**DOI:** 10.3779/j.issn.1009-3419.2020.02.07

**Published:** 2020-02-20

**Authors:** 文盛 周, 伟 张, 宝惠 韩

**Affiliations:** 1 200240 上海，上海交通大学医学院 Shanghai Jiao Tong University School of Medicine, Shanghai, 200240, China; 2 200030 上海，上海交通大学附属胸科医院，上海交通大学 Department of Pulmonary Disease, Shanghai Chest Hospital, Shanghai, 200030, China

**Keywords:** 表皮生长因子受体, 20外显子, 肺肿瘤, 插入突变, Epidermal growth factor receptor, Exon 20, Lung neoplasms, Insertion mutation

## Abstract

全球肺癌发病率及死亡率居全部恶性肿瘤的首位，与非小细胞肺癌（non-small cell lung cancer, NSCLC）相关的治疗研究也是近年来的研究热点。表皮生长因子受体-酪氨酸激酶抑制剂（epidermal growth factor receptor-tyrosinekinase inhibitors, EGFR-TKIs）的出现，给携带EGFR基因突变的NSCLC患者治疗带来巨大的转变。但携带EGFR基因的20外显子插入突变（exon 20 insertion mutation, ex20ins mutation）患者却是EGFR基因突变群体中的特殊人群，往往对EGFR-TKIs耐药。本文对*EGFR* ex20ins突变相关的研究进行回顾分析，归纳其特点、检测手段、治疗手段，对*EGFR* ex20ins突变有更加全面的认识，将为临床应用提供帮助，为患者带来更大的益处。

## Abstract (en)

Lung cancer has the highest morbidity and mortality among malignant tumors worldwidely. Targeted therapy related to non-small cell lung cancer (NSCLC) is the research hotspot in recent year. The emergence of epidermal growth factor receptor-tyrosine kinase inhibitors (EGFR-TKIs) has brought a huge change in the treatment of patients with *EGFR* mutation. The patients with *EGFR* exon20 insertion are specific cohort in NSCLC. Reviewing the clinical researches to *EGFR* exon20 insertion mutation positive NSCLC, as well as summarizing character, testing methods and treatment, will provide a help for clinical application, bringing more benefits for patients at the same time.

在全球范围内，肺癌是最常见的恶性肿瘤，而且是癌症死亡率居高不下的主要因素，2018年全球肺癌新发病例高达209万余例，肺癌造成的死亡人数高达176万例^[1]^。其中非小细胞肺癌（non-small cell lung cancer, NSCLC）在肺部恶性肿瘤中占绝大多数（80%）^[2]^。表皮生长因子受体（epidermal growth factor receptor, *EGFR*）基因突变是肿瘤形成主要的驱动基因之一^[3]^，携带*EGFR*基因突变患者占NSCLC患者总量的比率，在亚裔人群中及高加索人群中分别为30%-50%和10%-20%^[4-7]^。从2004年开始，NSCLC的EGFR突变及EGFR酪氨酸激酶抑制剂（EGFR-tyrosine kinase inhibitors, EGFR-TKIs）之间的关系被逐步报道^[8, 9]^。靶向药物的出现，使得NSCLC的治疗取得大的飞跃，是人类向将肺癌转化为慢性疾病的这一愿望的迈进。

在携带*EGFR*基因突变的NSCLC患者中，最常见的突变的19外显子的缺失突变（exon 19 deletions, 19del），其次是21外显子的点突变（L858R）。二者被认为是*EGFR*的经典突变，占了*EGFR*基因突变中的80%-90%^[10]^。而20外显子插入突变（exon 20 insertion mutation, ex20ins mutation）的数量仅次于19del及L858R，占有*EGFR*突变中的4%-12%^[11]^，但*EGFR* ex20ins突变并不像前二者那样对一二代EGFR-TKIs敏感，是*EGFR*突变中最常见的非敏感突变^[12-14]^。因而ex20ins突变的人群的治疗是目前临床医生在临床管理中面临的一大难题。

## 1 特点

### 1.1 高异质性

*EGFR*基因由7号染色体上的28个外显子组成，其中20号外显子负责转录E762-K823位置的氨基酸，其中包括由E762-M766氨基酸构成的C-螺旋以及由A767-V774氨基酸构成的环^[14]^（图 1）。根据COSMIC v89^[15]^中的数据提示，20外显子中最常见的插入突变位点为D770_N771insX，其次是V769_D770insX，这提示了C-螺旋后环结构可能是ex20ins突变的常见位置（图 2）。C-螺旋及环结构异常是引起组织异常生长，肿瘤形成的插入突变的常见位点^[11]^。据Riess等的研究，至今发现有超过60种独特的*EGFR* ex20ins形式，在其研究中最常见的*EGFR* ex20ins依次为D770_N771 > ASVDN、N771_P772 > SVDNP和N771_H773dupNPH，约占ex20ins突变的50%^[11, 16, 17]^。在众多的突变形式中，最为特殊的是763_764insFQEA。763_764insFQEA是目前发现ex20ins突变里的敏感突变^[14]^，约占EGFR 20外显子插入突变中的6%，是对一代EGFR-TKIs（代表药物为吉非替尼、厄洛替尼）二代EGFR-TKIs（代表药物为阿法替尼、达克替尼）都敏感的突变。

**1:**
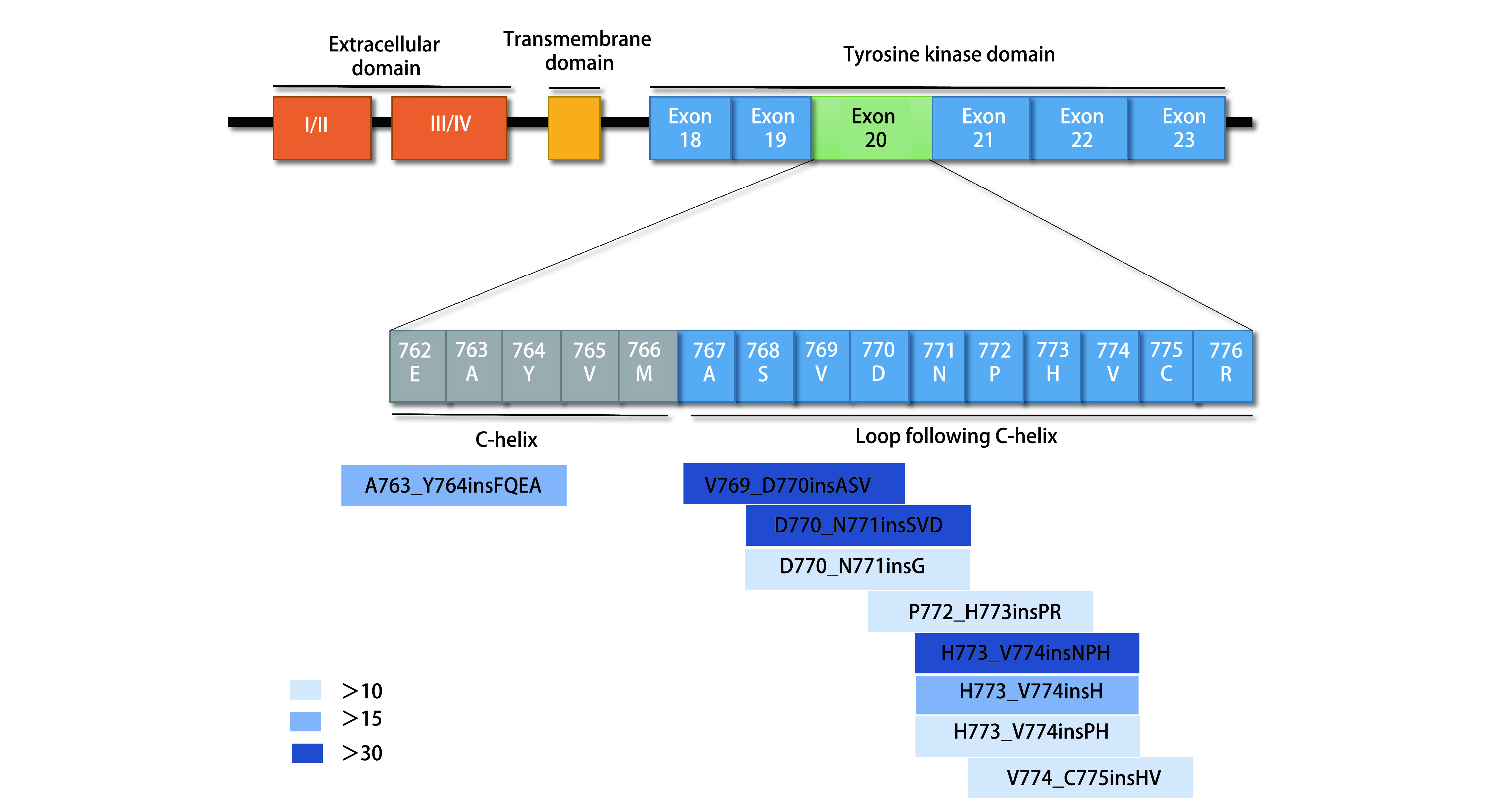
EGFR受体结构、*EGFR*基因20外显子编码的氨基酸及常见插入突变类型。数据来自于COSMICv89^[15]^（https://cancer.sanger.ac.uk/cosmic），图中所示突变类型为该数据库中经NSCLC、adenocarcinomas、EGFR20insertion筛选后样本量大于10的突变类型 Structure of EGFR, amino acids encoded by EGFR exon 20 and common types of insertion mutation. Data can be accessed on COSMIC v89^[15]^(https://cancer.sanger.ac.uk/cosmic). Mutation types whose sample size was over 10 were the results after filtering for NSCLC adenocarcinomas harboring exon 20 insertions

**2:**
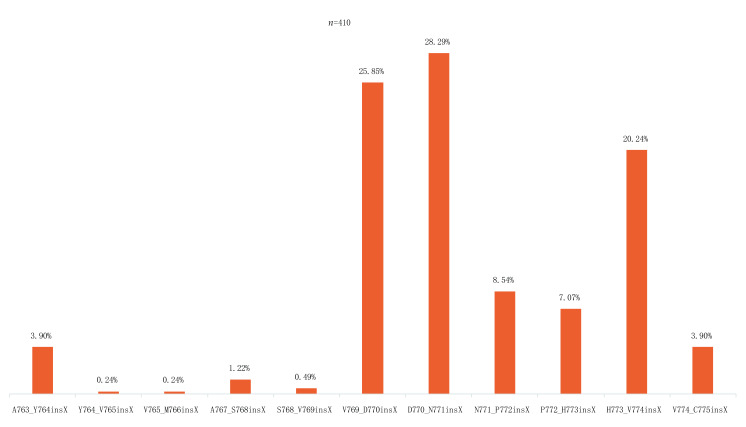
EGFR20外显子不同位置的插入突变频率。数据来自COSMICv89^[15]^（https://cancer.sanger.ac.uk/cosmic），经NSCLC、EGFR20insertion及in-framemutation筛选后获得（*n*=410），同一插入位点的不同插入类型统计为X，氨基酸突变跨度从A763到C775 EGFR exon 20 insertion mutation frequency at different amino acid positions. Mutation frequency distribution was calculated using COSMICv89^[15]^(https://cancer.sanger.ac.uk/cosmic)after filtering for NSCLC, adenocarcinomas harboring 20insertion and in-framemutation (*n*=410). Different insertion mutations occurring at the same amino acid position were collected as insX across a span of amino acids from A763 to C775

### 1.2 空间结构

EGFR在一级序列上包含配体结合的胞外区、单次跨膜区、激酶区和柔性的C末端片段。负责转录激酶区的*EGFR*基因中，经典的19del和L858R突变会引起激酶区构象改变，破坏EGFR失活状态的稳定性，使之向活化状态转变^[18-20]^。Ex20ins突变中的敏感突变A763_Y764insFQEA突变，插入的FQEA将C-螺旋向N末端移动，改变了β3-αC环的长度，导致I759的A替换。而β3-αC环是19缺失突变的经典位置，I759A改变与L858R和L861Q突变紧邻。由此看来，A763_Y764insFQEA相比于其他的*EGFR* ex20ins突变，更像L858R和19del^[14]^。有关携带EGFR A763_Y764insFQEA突变患者使用一代EGFR-TKI厄洛替尼后，对取得部分缓解（partial response, PR）疗效的报道，也提示了其与经典突变有着某种程度上的相似性^[21, 22]^。

EGFR 20外显子插入突变的其他类型，如D770_N771insNPG，在其酶动力学的研究中，D770_N771insNPG突变体与野生型EGFR有类似的结合模式和表观亲和力。该突变激活EGFR，并且没有显着降低其对ATP的亲和力，或增加其对EGFR-TKI的亲和力。其3D晶体结构揭示了，突变并未改变ATP结合口袋，而是在c-螺旋的末端形成的残基末端激活了酶构象。从结构来看，*EGFR* ex20ins突变体与吉非替尼的亲和力与野生型相似^[14, 18]^。这可能是携带*EGFR* ex20ins突变患者对一二代EGFR-TKIs不敏感的原因。

## 2 检测方法

针对肺癌相关靶向治疗分子的检测手段多种多样，临床上常用的技术有：实时聚合酶链式反应（real-time polymerase chain reaction, real-time PCR）、Sanger测序、免疫组化（immunohistochemistry, IHC）、免疫原位杂交（fluorescence *in situ* hybridization, FISH）、突变扩增阻滞系统（amplification refractory mutation system, ARMS）和二代基因测序（next-generation sequencing, NGS）。其中，应用于EGFR 20外显子插入突变的测序方法，Sanger测序的测序深度为20%^[23]^，real-time PCR的检测深度为1%^[24]^，而ARMS法和NGS可以检测到的等位基因频率可至0.1%^[25, 26]^。A763_Y764insFQEA是目前已知ex20ins突变中，被认为对EGFR-TKIs敏感的突变，因此明确突变的具体类型是决定临床治疗策略的关键之一。

ARMS法检测灵敏度和特异度高，信号可重复性好，自动化程度高，是目前应用最为广泛的*EGFR*基因突变的检测技术。但其扩增时使用的引物为针对已知突变设计，因而只能检测出标本中是否存在已知的突变类型，不能对未知突变类型进行探索。而NGS能对全基因组范围内的基因突变分析，包括基因突变、重排及拷贝数变异，并可发现未知突变类型，且敏感性明显高于Sanger测序，因此美国国立综合癌症网络（National Comprehensive Cancer Network, NCCN）指南中也将NGS列为推荐的检测技术。

IHC利用抗体对特定的分析物进行分析，具有很高的检测特异度，但由于抗体种类限制，可检测出突变类型也相对有限，目前临床上多运用于间变性淋巴瘤激酶（anaplastic lymphoma kinase, *ALK*）基因重组及*ROS1*基因重组的检测。FISH多用于拷贝数检测、拷贝数扩增及基因重组，因而多用于*c-MET*扩增突变、*ALK*基因重组及*ROS1*基因重组的检测。

## 3 治疗策略

作为*EGFR*基因突变中的罕见突变，较早的研究便指出，一代、二代EGFR-TKIs在*EGFR* ex20ins突变的患者中仅有局限的疗效^[16]^。对于携带*EGFR* ex20ins突变患者，除开特定的突变类型（A763_Y764insFQEA）对一二代的TKIs敏感，余多为EGFR-TKIs耐药突变，因此临床上优先的治疗策略是以铂类为基础的双药联合化疗，一二代的TKIs也不作为推荐药物使用。近年来，随着研究的不断深入，针对*EGFR* ex20ins突变的治疗药物层出不穷，如波齐替尼、Luminespib和TAK-788等许多药物都进入相应的临床试验中（表 1）。

**1:** 针对非小细胞肺癌*EGFR*基因20外显子插入突变阳性的临床试验 Clinical trials in *EGFR* exon 20 insertion positive NSCLC

Drugs	Clinical trial ID (s)	Targeting	Phase	Key results
Gefitinib/Erlotinib^[4, 27]^	Retrospective analysis of clinical studies	EGFR		PFS < 3 months (*n*=11, 25) RR 8%-25%
Erlotinib+Cetuximab^[43]^	NCT00895362	EGFR	Ⅰ	D770 > GY patient with 24.2+ months PFS
Afatinib^[34]^	NCT00525148 NCT00949650 NCT01121393	EGFR/HER2/HER4	Ⅱ	RR 8.7%, *n*=23, mPFS 2.7 months, mOS 9.2 months
Afatinib+Cetuximab ^[44]^	NCT03727724	EGFR	Ⅱ	Preliminary report, 3 out of 4 EGFR ex20ins with PR, 5.4 months PFS，ongoing
Dacomitinib^[35]^	NCT00225121	EGFR/HER2/HER4	Ⅰ	PR for 1 patient with D770delinsGY
Osimertinib^[38, 41]^	NCT03414814	EGFR	Ⅱ	mPFS 3.5 months (1.6 months-not reached), OS 12 months rate 56.3%
Tarloxotinib^[62]^	NCT03805841	EGFR/HER2	Ⅱ	Ongoing
Luminespib（AUY922）^[50]^	NCT01854034	Hsp90	Ⅱ	ORR 17% (*n*=29), mPFS 2.9 months (95%CI: 1.4-5.6), mOS 13 months (95%CI: 4.9-19.5)
TAK-788^[63]^	NCT02716116	EGFR/HER2 ex 20 ins	Ⅰ/Ⅱ	ORR 43%, *n*=28, mPFS 7.3 months, ongoing
Poziotinib^[48]^	NCT03066206 NCT04044170 NCT03318939	EGFR/HER2	Ⅱ	8-week ORR 58%, *n*=50，mPFS 5.6 months (abstract)
CLN-081^[53]^	NCT04036682	EGFR	Ⅰ/Ⅱa	Ongoing
DZD9008^[54]^	NCT03974022	EGFR/HER2	Ⅰ/Ⅱ	Ongoing
JNJ-372^[56]^	NCT02609776 NCT02609776	EGFR/cMet	Ⅰ	6/20 pts with Exon20ins had best timepoint response of PR (3 confirmed) (abstract)
Data can be accessed on https://clinicaltrials.gov/after filtering for exon 20 insertion. PFS: progression-free survival; PR: partial response; RR: response rate; ex20ins: exon 20 insertion.				

### 3.1 化疗

由于携带*EGFR* ex20ins突变NSCLC患者对一二代TKIs不敏感，甚至在接受化疗治疗中比一二代TKIs获益更多，客观缓解率（objective response rate, ORR）为58%-63%，无进展生存期（progression-free survival, PFS）为6.3个月^[5, 27]^。根据NCCN指南^[28]^，晚期非小细胞肺癌患者，*ALK*基因阴性或者是情况未知者、EGFR敏感性阴性或情况未知者细胞程序性死亡-配体1（programmed cell deathligand 1, PD-L1）表达 < 1%或情况未知者，都推荐以铂类为基础的双药联合化疗作为一线治疗方案。

### 3.2 TKIs单药治疗

以吉非替尼（Gefitinib）、厄洛替尼（Erlotinib）为代表的一代EGFR-TKIs，以可逆性共价键形式，与ATP竞争性结合EGFR，选择性地作用在携带有19del或L858R的NSCLC *EGFR*突变阳性的患者，ORR为62.1%-84.6%，PSF为9.2个月-10.8个月。相比而言，携带ex20ins突变的患者，几乎对一二代的EGFR-TKIs耐药，缓解率（response rate, RR）为0%-11%，PFS为2个月-3个月^[29-33]^。

以阿法替尼（Afatinib）、达克替尼（Dacomitinib）为代表的二代EGFR-TKIs，以不可逆性共价键形式与EGFR受体特异性的残基（C797）结合。在现有的为数不多的关于EGFR 20外显子插入突变的临床实验数据中提示，阿法替尼在携带*EGFR* ex20ins突变患者的疗效并不佳，仅有8.7%的RR（*n*=23），2.7个月的中位无进展期（median progress-free survival, mPFS），9.2个月的中位总生存期（median overall survival, mOS）^[34]^。而达克替尼^[35]^的临床试验中，也仅有1例患者取得PR疗效（*n*=6）。

以奥希替尼（Osimertinib）为代表的三代EGFR-TKIs，针对NSCLC中一代EGFR-TKIs耐药突变T790M患者，选择性地与EGFR的C797处氨基酸残基共价结合，抑制其磷酸化和下游信号底物蛋白激酶B（protein kinase B, PKB）和细胞外调节蛋白激酶（extracellular signal-regulated kinase, ERK）的磷酸化^[36]^。在体外实验中，奥希替尼在携带*EGFR* ex20ins突变的细胞株中，表现出抑制肿瘤细胞生长的活性，和比阿法替尼稍弱的结合力。在肿瘤异种移植模型中，奥希替尼在160 mg/d的剂量下，显示出了一定的抗肿瘤疗效^[12, 37-39]^。在现有的小样本的研究^[40]^中，报道6位携带*EGFR* ex20ins突变患者使用奥希替尼后，肿瘤均得到了控制，ORR 100%，mPFS为6.2个月。由此可见，奥希替尼在该人群中存在一定的疗效，但由于样本量过小，疗效仍需要进一步研究及探索。而以奥希替尼作为携带*EGFR* ex20ins患者治疗手段的Ⅱ期临床试验（NCT03414814）数据报道^[41]^，mPFS为3.5个月（1.6个月-未达到），1年总生存率（overall survival, OS）为56.3%，目前研究仍在进行中。

### 3.3 TKIs联合西妥昔单抗

EGFR-TKIs联合西妥昔单抗（Cetuximab）或许是可行的治疗方案之一。与单独使用EGFR-TKIs相比，EGFR-TKIs和西妥昔单抗的联合使用显示，可以更彻底地消耗磷酸化和总的EGFR，进而在患有L858R/T790M厄洛替尼耐药的小鼠中引起近乎完全的肿瘤消退^[42]^。在厄洛替尼联合西妥昔单抗的临床试验中，1例患者D770 > GY取得了3.5年PFS^[43]^。而在阿法替尼联合西妥昔单抗临床试验中^[44]^，根据早期的报告示，四位携带EGFR 20外显子插入突变患者中的3例取得PR疗效，mPFS为5.4个月。近期Fang等^[45]^报道的个案中，奥希替尼联合西妥昔单抗二线治疗*EGFR* ex20ins突变NSCLC有效，目前该患者PFS大于5个月。目前为止，这些临床数据都提示了TKIs联合西妥昔单抗对*EGFR* ex20ins突变具有一定的效力，但是不同的插入类型对该方案的敏感性及疗效是否存在差异需要进一步的临床试验来探索。

### 3.4 波齐替尼

波齐替尼（Poziotinib，前身为HM781-36B），一种选择性与EGFR和HER2不可逆共价结合的阻滞剂^[46]^。在体外研究中，Cha等评估了HM781-36B的治疗潜力，发现HM781-36B在多种EGFR和HER2依赖性肿瘤异种移植模型中显示处优异的功效^[47]^。体外实验中的优异表现，为当下进行中的波齐替尼的Ⅱ期临床研究（NCT03066206）打下理论基础。在该研究中，研究者使用3D建模技术，发现20外显子的突变改变了药物结合区的空间大小，进而阻滞了体积较大的抑制剂的结合，而波齐替尼则是利用其体积较小、灵活性好等优势，克服了突变带来的空间阻滞作用。目前，波齐替尼的Ⅱ期临床试验中，相关数据报道^[48]^，8周时的ORR为58%（95%CI: 40.9-73.0），疾病控制率（disease control rate, DCR）为90%（95%CI: 76.3-97.2），mPFS为5.6个月（95%CI: 5.06-NA）。

### 3.5 Luminespib

Luminespib是一种热休克蛋白90阻滞剂（前身为AUY922）。热休克蛋白90是一种分子伴侣，是维持和协助蛋白质折叠稳定性所需要的细胞蛋白，其中包括驱动肿瘤发生相关的受体和信号通路成分^[49]^。在其基础研究结果提示，热休克蛋白阻滞剂可通过热休克蛋白90相关分子系统，降解*EGFR* ex20ins及其下游信号通路，最后诱导细胞凋亡。Luminespib的Ⅱ期临床试验结果^[50]^表明ORR为17%（*n*=29），这相比于阿法替尼所带来的8.7% RR，是明显获益的，但是短暂的PFS为2.9个月，OS为13个月使得Luminespib在临床治疗中能否为患者带来更大的治疗效益，需要进一步得临床研究来探索。

### 3.6 TAK-788

TAK-788（前身为AP32788）被设计为选择性作用于20外显子插入突变体（包括EGFR和HER2）的阻滞剂^[51]^。通过Ba/F3细胞株对AP32788的活性评估，AP32788能抑制EGFR的14种突变类型及HER2的6种突变类型。在其进行中的Ⅰ期/Ⅱ期临床试验中（NCT02716116），TAK-788显示出了不错的疗效。在28例患者中得到疗效评估PR的患者有14例，ORR为43%，mPFS为7.3个月^[52]^。

### 3.7 新型TKIs

CLN-081（前身为TAS6417）是一种新型的EGFR抑制剂，针对*EGFR* ex20ins突变。在体外的Ba/F3细胞株实验中，CLN-081表现出相比于野生型，对*EGFR* ex20ins更强的抑制。与此相关的肿瘤异种移植模型实验中，CLN-081的应用使得肿瘤明显消退^[53]^。其Ⅰ期/IIa期临床试验（NCT04036682）正在进行中。

DZD9008为针对EGFR及HER2 ex20ins突变的口服TKI制剂。DZD9008在表达EGFR L858R、Exon19del、L858R/T790M，各种Exon20ins或罕见突变的肿瘤细胞系中，以半数抑制浓度（50% inhibitory concentration, IC_50_）1 nmol/L-22 nmol/L下调磷酸化的EGFR，而在表达野生型EGFR的肿瘤细胞中调节磷酸化的EGFR的作用较弱，IC_50_ > 80 nmol/L。在细胞增殖测定中以半数生长抑制浓度（50% growth inhibition concentration, GI_50_）1 nmol/L-60 nmol/L，抑制了携带*EGFR* ex20ins突变不同类型的肿瘤细胞的增殖。在CDX及PDX模型中，DZD9008介导了剂量依赖性肿瘤生长抑制的关系^[54]^。进一步的临床获益，则需要临床试验来探索，目前相关Ⅰ期/Ⅱ期临床试验正在进行中（NCT03974022）。

### 3.8 JNJ-372

JNJ-372，一个新型的EGFR-cMET特异性双抗体^[55]^，可阻断原发性及继发性*EGFR*突变体和cMET通道。JNJ-372通过阻断配受体结合来抑制激活，其次通过降解受体诱导其失活，最后利用通过Fc段介导抗体依赖的细胞介导的细胞毒性作用（antibody-dependent cell-mediated cytotoxicity, ADCC）对肿瘤细胞进行杀伤。体外肿瘤异种移植模型实验中，与三代EGFR-TKI合用，取得持续完全的肿瘤消退。且在食蟹猴的实验中未出现明显的不良事件。其近来的Ⅰ期临床试验数据更新显示^[56, 57]^，20例入组的携带*EGFR* ex20ins突变的患者中，有6例得到最佳疗效为PR。更多进一步的数据则需要等待临床试验的进行。

### 3.9 免疫治疗

免疫治疗在NSCLC患者中进行的临床试验如火如荼地进行。根据NCCN指南（v5.2019），PD-L1 > 1%且EGFR、ALK阴性或者未知的患者推荐使用帕博利珠单抗（Pembrolizumab）等免疫制剂。但指南中关于*EGFR*非敏感突变患者的使用却未见说明。Yamada等^[58]^的回顾性研究中，携带*EGFR*罕见突变病人或者是敏感突变耐药后，使用PD-1/PD-L1受体抑制剂作为后线治疗，罕见突变病人较敏感突变病人获益更大（疾病控制率57% *vs* 7%，*P* < 0.01）。但在暂时缺乏针对*EGFR* ex20ins突变的免疫治疗的临床试验。PD-1/PD-L1受体抑制剂对*EGFR* ex20ins突变患者是否能带来相比于化疗更大的获益，这需要进一步的临床研究来证实。

## 4 结语

EGFR-TKIs的药物毒性来自于正常组织中WT EGFR的限制，但由于*EGFR*突变的存在，肿瘤组织对EGFR抑制剂的敏感性增高，使得TKIs成为具有临床效益的药物。晶体结构上来看，*EGFR* ex20ins突变不同于经典的敏感突变（19del和L858R），而更像WT型^[27, 59]^，由于一二代EGFR-TKIs对ex20ins突变的选择性并不高，增加药物浓度带来的毒性反应的限制，使得药物在肿瘤内未能达到有效的浓度，这或许是临床结果欠佳的原因之一^[60, 61]^。随着波齐替尼、TAK-788和JNJ-372等药物的问世与临床试验的进行，携带*EGFR* ex20ins的NSCLC患者在治疗上的选择会越来越多。奥希替尼3.5个月的mPFS，波齐替尼5.6个月的mPFS，TAK-788 7.3个月的mPFS，都预示着*EGFR* ex20ins突变的前路只会越来越宽。但同时，这些新型药物使用的后续问题也是未知而且需要关注的，这些药物长期使用后是否会引起耐药突变，耐药的机制及应对的策略，这都是至关重要的。在*EGFR* ex20ins突变中，突变位点可在c-螺旋区、c-螺旋后环结构区，具有高异质性。不同的突变位点对药物的敏感性可能不同，因而这需要对受体蛋白结构的进一步了解，进而有助于药物的研发及个体化治疗。随着对*EGFR* ex20研究的不断深入，会有更多针对该罕见突变的药物出现，为患者带来更多的获益。
